# Large terahertz photovoltaic effect enhanced by phonon excitations in ferroelectric semiconductor SbSI

**DOI:** 10.1126/sciadv.adw9796

**Published:** 2026-03-06

**Authors:** Yoshihiro Okamura, Guang-Yu Guo, Yoshio Kaneko, Masao Nakamura, Masato Sotome, Naoki Ogawa, Masashi Kawasaki, Yoshinori Tokura, Youtarou Takahashi

**Affiliations:** ^1^ Department of Applied Physics and Quantum Phase Electronics Center, The University of Tokyo, Tokyo 113-8656, Japan.; ^2^ Department of Physics and Center for Emerging Material and Advanced Devices, National Taiwan University, Taipei 10617, Taiwan.; ^3^ Physics Division, National Center for Theoretical Sciences, Taipei 10617, Taiwan.; ^4^ RIKEN Center for Emergent Matter Science (CEMS), Wako 351-0198, Japan.; ^5^ Department of Physics, Graduate School of Science, Tohoku University, Sendai 980-8578, Japan.; ^6^ RIKEN Baton Zone Program (BZP), Wako 351-0198, Japan.; ^7^ Department of Materials Engineering, The University of Tokyo, Tokyo 113-8656, Japan.; ^8^ Research Center for Advanced Science and Technology, The University of Tokyo, Komaba, Tokyo 153-8904, Japan.; ^9^ Tokyo College, The University of Tokyo, Tokyo 113-8656, Japan.

## Abstract

Quantum geometry of Bloch electron in crystalline solids produces various exotic quantum phenomena. The shift current photovoltaic effect driven by the photo creation of quasiparticle is one such emerging example that enables the conversion from terahertz photon into dc charge current with absence of dissipative photocarrier. Despite wide-ranging potential applications, however, the fundamental nature of terahertz photovoltaic response has remained elusive. Here, we show the large photocurrent generation driven by terahertz phonons (<10 milli–electron volts) in ferroelectric semiconductor SbSI with the electronic bandgap of 2.3 electron volts. Zero-bias terahertz photocurrent is found to be resonantly enhanced by optical phonons. Its generation efficiency is larger than that for the direct interband transition and is comparable to the electronic shift current in Weyl semimetal TaAs. The theoretical scaling law of terahertz shift current and first-principles calculation reasonably explain these observations. The present work establishes the universality and high efficiency of phonon-driven shift current, opening the pathway to terahertz technology based on quantum geometry.

## Teaser

Quantum geometry of matter enables gigantic terahertz shift current generation through photo-creation of optical phonons.

## INTRODUCTION

Terahertz technology is envisioned for the use of broadband wireless communication and various sensing applications (*1*). Among many issues to be resolved for the future terahertz technology, the conversion technique from terahertz photon into electric current/voltage is much less developed than the visible or near-infrared region (*2*, *3*), although it is indispensable for the integration of terahertz photonic and electronic circuits. Various types of terahertz detectors have been suggested up to date, such as Golay cells, bolometers, pyroelectric sensors, Schottky diode, and two-dimensional (2D) material–based device (*1*–*5*). However, there is still no established principle that realizes terahertz detection exhibiting broadband, high-speed, and low-noise photoresponsivity at room temperature, while operating without any device fabrication, external bias, or circuit integration, boosting the importance of fundamental research on the terahertz photovoltaic effects.

Quantum geometrical aspects of electronic dynamics in solids stimulate the recent advances in the theoretical prediction and experimental realization of nonlinear transport and optical phenomena (*6*–*9*). The shift current mechanism for bulk photovoltaic effect (BPVE), which enables photon-to-dc current conversion in single-phase noncentrosymmetric materials, is one notable example (*10*–*18*). The instantaneous real-space shift of position of electronic wave packet upon the interband optical transition, which is described by geometrical shift vector, generates the steady-state dc photocurrent. However, the photon energy required for conventional BPVE is limited to the electronic excitation above bandgap energy on a typical energy scale of electron volt, i.e., visible and near-infrared region. One important theoretical implication of the shift current mechanism is that the generated photocurrent is driven by electric dipole moment of photoexcited electron-hole pairs and is independent of the creation and drift of photocarriers. Therefore, photocreation of any charge neutral quasiparticles potentially gives rise to the photovoltaic effect if these quasiparticles accompany electric dipole moment through coupling with electronic states (*19*–*23*). Namely, in contrast to the conventional BPVE via electronic excitation, the low-energy quasiparticles far below the bandgap energy can be used for the terahertz photovoltaic effect through this extended shift current mechanism (*20*, *22*, *23*). To be more specific, the resonant excitation of phonon or magnon leads to the instantaneous change of electronic polarization characterized by Berry connection through the electron-phonon coupling or spin-orbit interaction, resulting in the generation of the shift current. The terahertz dc photocurrent generation via quasiparticle creation are experimentally demonstrated for the soft phonon of archetypal ferroelectric BaTiO_3_ (*22*) and for the spin excitations of multiferroics (*23*). This extended shift current of quasiparticles has many technological advantages due to the geometrical origin, such as ultrafast responsivity, less-dissipative energy flow, and low-energy divergence of conversion efficiency (*17*, *18*). However, despite these practical advantages for terahertz photonics, the fundamental nature of terahertz photovoltaic effect has been scarcely investigated including the quantitative evaluation of photovoltaic performance, spectral response, and mode dependence.

Here, we show the large terahertz phonon-induced shift current in displacive-type ferroelectric semiconductor SbSI, which is known to show the large electronic shift current in visible region (*16*–*18*). In terahertz region, the strong terahertz absorption from soft phonon emerges because of the ferroelectric instability in addition to the normal infrared-active phonon. Accordingly, SbSI is a promising material for the enhanced terahertz shift current generation. We observe the appreciable zero-bias dc photocurrent by the direct excitation of soft phonon and normal optical phonon. By using the terahertz photocurrent spectroscopy, we reveal that the nonlinear terahertz conductivity is resonantly enhanced upon each phonon energy and its magnitude critically depends on the mode character. These observations are explained by the first-principles calculation incorporating the extended shift current mechanism. The obtained terahertz photon-to-dc conversion efficiency for soft phonon is substantially large, being comparable to the large shift current for electronic interband excitation in Weyl semimetal TaAs (*15*).

## RESULTS

### Ferroelectric semiconductor SbSI

**Fig. 1.**
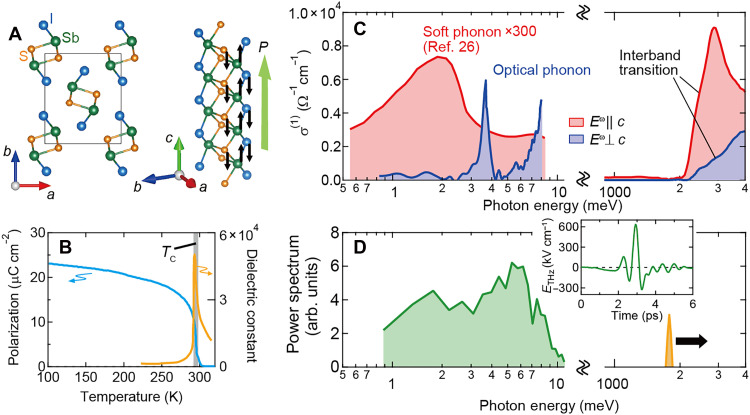
Displacive-type ferroelectric SbSI and terahertz optical phonons. (**A**) The crystal structure of the ferroelectric semiconductor SbSI. The left panel shows the cross-sectional view in the *ab* plane. The black arrows in the right panel denote the direction of atomic displacements upon the ferroelectric transition. (**B**) Temperature dependence of the spontaneous polarization (light blue curve) and dielectric constant at 1 kHz [orange curve, reproduced from (*24*)]. The gray bar represents the ferroelectric transition temperature *T*_C_. (**C**) The optical conductivity spectra σ^(1)^ for *E*^ω^||*c* (red curve) and *E*^ω^⊥*c* (blue curve) in the wide energy range. The σ^(1)^ for *E*^ω^||*c* in terahertz region is calculated from (*26*). (**D**) The spectral amplitude of the terahertz electric field used in the present study (green curve). (inset) Time waveform of the terahertz light pulse. The yellow hatched region schematically illustrates a typical power spectrum of irradiated visible and near-infrared light for electronic excitation. The scan range of photon energy is indicated by the black arrow.

The crystal structure of SbSI in the paraelectric phase belongs to space group *Pnam* and turns into *Pn*2_1_*a* in the ferroelectric phase at *T*_C_ ~ 295 K (Fig. 1A) (*24*, *25*); the S- and Sb-ions are displaced from the paraelectric positions, resulting in spontaneous polarization of *P* ~ 25 μC cm^−2^ along the *c* axis (Fig. 1B). Reflecting the displacive-type transition nature, the transverse optical phonon with the broad spectral width shows substantial frequency lowering toward the *T*_C_ as observed in the *c*-polarized linear optical conductivity spectrum σ^(1)^(ω) (Fig. 1C, red curve and see fig. S1) (*26*). Below the *T*_C_, this soft phonon is frozen but still retains the peak in the terahertz region, leading to the strong optical absorption for *c*-polarized terahertz light. On the other hand, a sharp peak of normal optical phonon is discerned around 3.5 meV for σ^(1)^(ω) polarized perpendicular to the *c* axis (Fig. 1C, blue curve; see also fig. S2); the increase of σ^(1)^ above 6 meV is a lower-lying tail of other higher-lying infrared-active optical phonons. The peak energy of the phonon mode at 3.5 meV shows little temperature dependence, and the spectral width is much sharper than that of the soft phonon, both of which suggest that this phonon mode shows little correlation with ferroelectric transition.

SbSI is also known to exhibit versatile photoinduced phenomena (*27*, *28*). In particular, the BPVE upon electronic excitation in visible region has been thoroughly studied by using various experimental techniques in recent years (*16*–*18*, *29*, *30*). This compound respectively has the indirect and direct bandgap of 2.15 and 2.3 eV, as indicated by the steep increase of σ^(1)^ around ~2.3 eV (Fig. 1C). The large zero-bias photocurrent for the electronic excitations is ascribed to the shift current mechanism. The spectral response of photocurrent is also established with the help of the optimization of experimental conditions such as electrical circuit and electrode material (*29*, *30*), being the useful reference for the quantitative discussion of terahertz photovoltaic performance in the present study.

### Terahertz BPVE

**Fig. 2.**
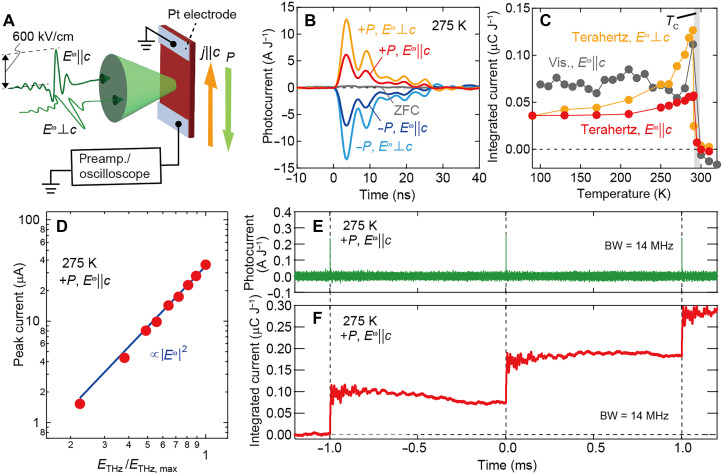
Phonon-induced BPVE. (**A**) The schematic illustration of the experimental setup. The black double arrow represents the typical peak terahertz electric field ~600 kV/cm. (**B**) Pulsed photocurrent for *E*^ω^||*c* and *E*^ω^⊥*c* in the single-domain ferroelectric states (+*P* and –*P*) at 275 K. The gray curve represents the photocurrent for the multidomain state after zero field cooling (ZFC). (**C**) Temperature dependence of the photocurrent integrated over time and normalized by irradiated pulse energy. The red and orange circles denote the data of terahertz photocurrent for *E*^ω^||*c* and *E*^ω^⊥*c*, respectively. The gray one corresponds to the electronic excitation (1.95 eV), as reproduced from (*16*). The gray bar represents the ferroelectric transition temperature *T*_C_. (**D**) Terahertz electric field dependence of the peak value of photocurrent pulse (red circles). The blue line represents the fitting line using |*E*^ω^|^2^. (**E** and **F**) Transient photocurrent response for the long-time scale (E) and the corresponding integrated current (F) (see Materials and Methods for the details of experimental conditions).

The BPVE is generally described by the second-order nonlinear optical conductivity tensor σ^(2)^*_ijk_*, where *ijk* = *caa*, *cbb*, and *ccc* are allowed for the *c*-direction photocurrent in the ferroelectric SbSI from the viewpoint of symmetry. Note that we define *j_i_ =* σ^(2)^*_ijk_*(ω)*E_j_E_k_*, where *j_i_* and *E_j_* (*E_k_*) respectively represent the photocurrent density along the *i* axis and the electric field of light along the *j* (*k*) axis, and that the sign of photocurrent opposite to the *P* direction is set to be positive. For the photocurrent measurement, we used the intense terahertz light pulse generated by tilted-pulse front method (*31*), whose broad spectral range (1 to 7 meV) is suitable for the direct excitation of two types of terahertz phonons in SbSI (Fig. 1, C and D). The photocurrent signal as a function of time was measured by using an oscilloscope (Fig. 2A).

Figure 2B shows the time profiles of photocurrent under the terahertz pulse excitation. We observed the appreciable zero-bias photocurrent pulses in the single-domain ferroelectric state for both light polarizations (*E*^ω^||*c* and *E*^ω^⊥*c*) but never for the zero-field-cooling state with zero net *P* (gray curve). The sign of photocurrent is reversed by the reversal of the direction of *P*. In contrast, the photocurrent signal abruptly disappears in the centrosymmetric paraelectric phase above the *T*_C_ (Fig. 2C). These observations demonstrate that the photocurrent is concomitant with the spontaneous inversion symmetry breaking, in accord with the symmetry requirement for the BPVE. The magnitude of the photocurrent is proportional to the square of the terahertz electric field (Fig. 2D), which is also consistent with the BPVE. The steep increase of terahertz photocurrent toward the *T*_C_ in Fig. 2C is ascribed to the critical divergence of susceptibility [nonlinear optical conductivity σ^(2)^] near the phase transition (*22*, *23*). The low-energy terahertz response probably tends to be more sensitive to the critical divergence, which dramatically enhances the terahertz photocurrent for *E*^ω^⊥*c* by a factor of almost 3.5.

It should be emphasized that the observed photocurrent pulse does not show any sign change as the function of time (Fig. 2B), signaling the dc photocurrent generation as expected for BPVE. Note that the time resolution of a few nanoseconds for photocurrent signal is determined by the bandwidth of electric circuit, and the oscillatory waveform following the main peak is caused by the ringing of the electric circuit used for the photocurrent detection (see Materials and Methods). The dc photocurrent generation is further corroborated by looking into the long-time photocurrent response over several excitation pulses (Fig. 2E). Corresponding to the repetition rate of 1 kHz of terahertz pulse, the sharp photocurrent pulse is observed every 1 ms. The time integration of the current signal shows the step function–like increase at each pulse (Fig. 2F). Since only the dc photocurrent gives rise to the finite charge accumulation by the time integration, these observations conclusively evidence that the genuine steady-state photocurrent is created by the terahertz light irradiation, i.e., terahertz photon is converted to the dc current by BPVE. Possible contributions from photothermally induced pyroelectric current, optical rectification, and piezoelectric effect, which cannot generate finite integrated charge in this repetitive experiment, are negligible. We note that since the terahertz excitation is never accompanied by the photocarrier generation in contrast to the conventional photovoltaic effect due to electronic excitation, the open-circuit voltage, which is usually caused by the photocarrier generation, should be substantially suppressed for terahertz BPVE; this observation is left for future studies.

### Quantitative evaluation of σ^(2)^

Having established the terahertz BPVE, we next discuss the response function characterizing photoresponsivity, σ^(2)^(ω), which is given as (*12*)

$$\sigma_{\mathrm{ijj}}^{\left(2\right)}\left(\omega \right)=\frac{1}{2}\sqrt{\frac{\varepsilon_{0}}{\mu_{0}}}\cdot \frac{J_{i}\left(\omega \right)}{I_{\text{abs}}}\cdot \frac{\alpha_{j}\left(\omega \right)}{w}$$ (1)

ε_0_ and μ_0_ respectively represent the dielectric constant and permittivity of vacuum; *J_i_*(ω) and *I*_abs_ respectively represent the photocurrent along the *i* axis and absorbed light power; α*_j_*(ω) and *w* respectively represent absorption coefficient for the light polarized along the *j* axis and width of the sample surface. The photon energy–dependent *J_i_*(ω) was measured by monochromatizing the broad power spectrum of incident terahertz pulse by using band pass filters (figs. S3 and S4). In the present experimental condition, the measured photocurrent, *J_i_*(ω) in Eq. 1, for the pulsed light with a few-picosecond duration (see inset to Fig. 1D) is quantitatively underestimated because the photocurrent generated by terahertz pulse should be attenuated because of the limitation of bandwidth of electrical circuit used for photocurrent detection. To qualitatively deduce the magnitude of *J_i_*(ω), we calibrated the measured photocurrent by taking into account the attenuation rate of electric circuit (for more details, see note S2 and fig. S5).

**Fig. 3.**
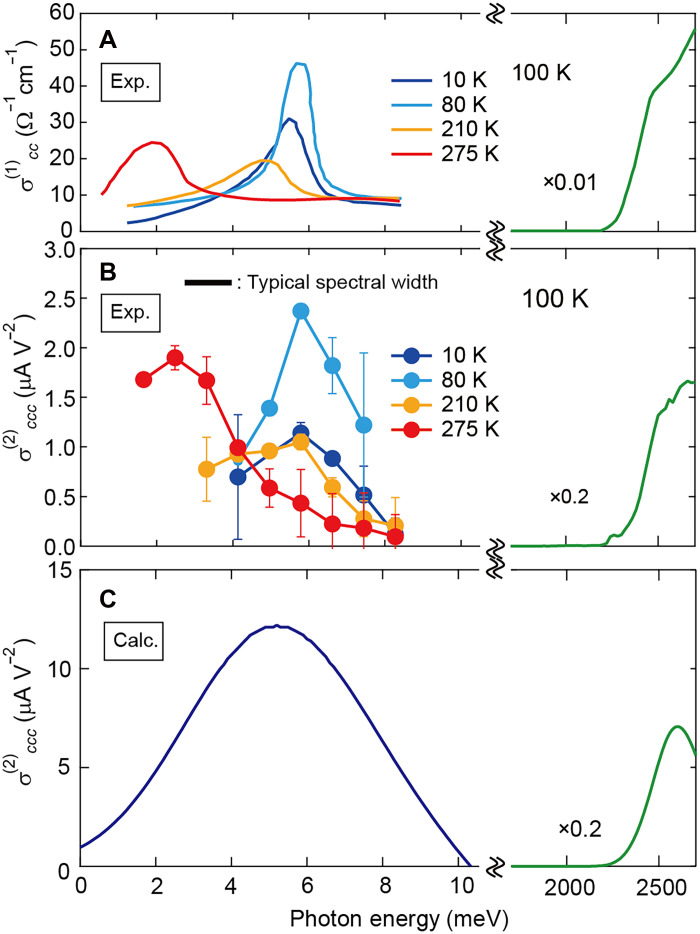
Linear and nonlinear optical conductivities. (**A**) The linear optical conductivity polarized along the *c* axis, σ^(1)^*_cc_*. The σ^(1)^*_cc_* in terahertz and visible regions are calculated from (*26*) and (*18*), respectively. (**B**) The second-order nonlinear optical conductivity spectrum σ^(2)^*_ccc_* obtained from the experiment. The σ^(2)^*_ccc_* in visible region is calculated from (*18*). The error bars arise from the deviation between Lorentzian fit and experimental ε; within this uncertainty, the phonon softening can be well identified (see also fig. S1)*.* The experimental uncertainty of σ^(2)^ estimated using the statistical error of the photocurrent pulses (Fig. 2E) is 3.4%, which is smaller than the size of each symbol. The black horizontal bar represents the typical half width at half maximum of the irradiated terahertz power spectrum (see fig. S3). (**C**) The σ^(2)^*_ccc_* spectra from the shift current mechanism calculated using the density functional theory (see note S3).

Figure 3 (A and B) summarizes the linear [σ^(1)^] and nonlinear [σ^(2)^] optical conductivity spectra for both soft phonon excitation in terahertz region and the electronic excitation in visible region. The terahertz σ^(1)^ and σ^(2)^ spectra show the clear resonance structures with similar temperature change; the peak shift in σ^(2)^ well coincides with the shift of soft-phonon energy observed in the σ^(1)^ spectra. This observation clearly demonstrates that the photocreation of phonon gives rise to the terahertz BPVE as expected from the extended shift current mechanism. We can further verify this by calculating the σ^(2)^ from the first-principles calculation incorporating the extended shift current mechanism (Fig. 3C; for the detail of calculation, see note S3). Theoretical spectra at zero temperature well reproduce the essence of experimental σ^(2)^ spectra (see also fig. S6), emergence of the positive-sign resonance on the soft phonon, while the overall magnitude of σ^(2)^ tends to be overestimated in the calculation possibly because partial deexcitation of photoexcited phonons is not included.

We emphasize that the resonance of σ^(2)^ in terahertz region is observed also for the light polarization perpendicular to the *c* axis, indicating the shift current generation from the normal infrared-active phonon (fig. S7). Accordingly, the phonon-induced shift current is not limited to the soft phonon in displacive-type ferroelectrics but would be universal for any infrared-active optical phonons in various polar systems. On the other hand, the peak magnitude of σ^(2)^ for the soft phonon (Fig. 3B) is two orders of magnitude larger than that for the normal optical phonon (fig. S7), which is well reproduced by the first-principles calculation (fig. S8). This clear mode dependence is ascribed to the strength of electron-phonon coupling that plays the important role in magnitude of σ^(2)^ for the phonon-induced shift current (*22*); the soft phonon is generally known to be strongly coupled to the electronic polarization, as suggested by the large spectral weight in σ^(1)^ through transfer of oscillator strength from electronic excitations.

We note that the light polarization anisotropy of σ^(2)^ is much larger than that of σ^(1)^ (Fig. 3 and fig. S7). We derived the theoretical formulation of the phonon-driven shift current based on the 1D Rice-Mele model, which consists of two inequivalent sites, coupled to phonons (*22*). Within this minimal model, we separately calculated the conductivities σ^(1)^ and σ^(2)^ and found that they are related by

$$\sigma^{\left(2\right)}=\frac{\mathrm{eR}}{\hbar \omega }\sigma^{\left(1\right)}$$ (2)

where *R* corresponds to the phonon-induced shift vector, and ℏω represents the photon energy. Thus, the magnitude and spectral characteristics of σ^(2)^ are governed by the product of σ^(1)^ and *R*. Since *R* represents the real-space shift of the electronic distribution induced by phonon excitation, its behavior is influenced by complex band dispersions, deformation pattern of lattice, and the resulting electron–phonon interactions, showing a strong dependence on the phonon mode. Consequently, both σ^(1)^ and *R* show the mode dependence, resulting in larger polarization anisotropy in σ^(2)^ than that either in σ^(1)^ or *R*.

## DISCUSSION

### Terahertz photovoltaic performance and DC photoresponsivity

We compare the presently observed photovoltaic performance with other compounds. When the penetration depth of the light is small enough as compared to the sample thickness, the performance of the BPVE is quantified by the Glass coefficient, which is written as (*32*)

$$G_{\mathrm{ijj}}=\frac{2\sigma_{\mathrm{ijj}}^{\left(2\right)}}{\alpha_{j}}\sqrt{\frac{\mu_{0}}{\varepsilon_{0}}}$$ (3)

**Fig. 4.**
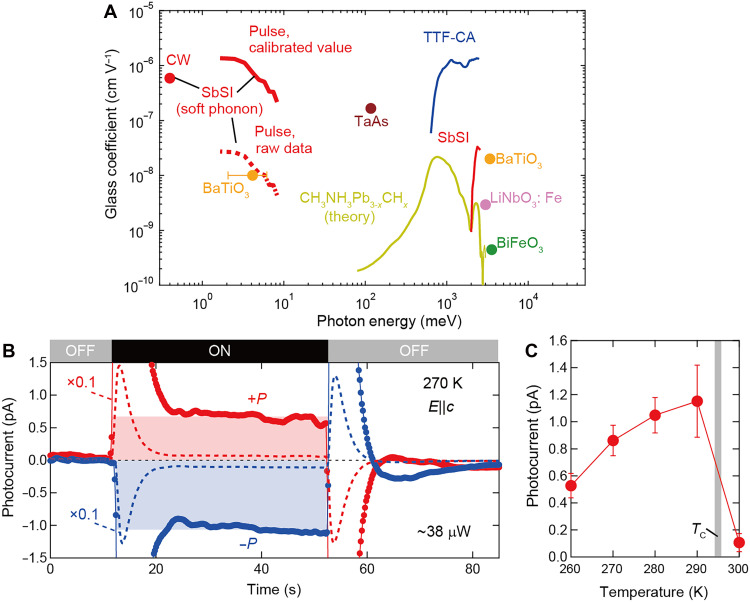
Terahertz photovoltaic performance and DC photoresponsivity. (**A**) The Glass coefficient of various polar materials in the wide energy range. The calibrated value for SbSI is calculated by considering the attenuation of pulsed photocurrent in the electrical circuit as discussed in note S2 (red solid curve). For the CW excitation at 100 GHz (~0.42 meV), calibration is not required. For comparison with uncalibrated data of terahertz photovoltaic effect for BaTiO_3_ (orange circle in terahertz region), the raw spectrum of Glass coefficient for SbSI is displayed (red dashed curve). (**B**) Temporal profile of photocurrent under the CW subterahertz irradiation polarized along *c* axis. The light is irradiated for “ON” state and not for “OFF” state. The red and blue symbols represent the photocurrent response for +*P* and −*P* states, respectively. (**C**) Temperature dependence of steady-state photocurrent for 100-GHz excitation.

Thus, we calculate the terahertz Glass coefficient for *E*^ω^||*c* from σ^(2)^ at 275 K (red curve, Fig. 4A). The resonant enhancement of Glass coefficient is observed around the soft-phonon resonance, reaching 1.36 × 10^−6^ cm V^−1^ at 1.65 meV (0.4 THz). This value is larger than the Glass coefficient of SbSI in the visible region and comparable to that of the materials known to show large shift current generation, such as organic ferroelectric tetrathiafulvalene–*p*-chloroamphetamine and of polar Weyl semimetal TaAs (*10*, *14*, *15*, *18*, *22*, *33*–*35*). This marked value in terahertz region can be explained by the theoretically suggested scaling of σ^(2)^ in addition to the critical divergence near the *T*_C_ discussed above; the magnitude of σ^(2)^ for the phonon-induced shift current involves a factor being inversely proportional to the photon energy and shows the low-energy enhancement (*22*). Thus, the soft phonon, which hosts the low resonance energy and strong electron-phonon coupling, has the substantial potential for high conversion efficiency of terahertz photon to dc current as demonstrated in the present study.

Motivated by the large terahertz Glass coefficient, as an experimental proof of concept toward terahertz detector applications, we finally demonstrate the dc photocurrent response under the continuous-wave (CW) sub-terahertz illumination polarized along *c* axis, with frequency of 96.47 GHz and irradiated power as low as 38 μW (see also Materials and Methods). We observe that an appreciable transient current arises from the pyroelectric response due to the rapid increase in sample temperature immediately after photoexcitation, which is followed by a steady-state dc photocurrent, as indicated by the red and blue hatched regions in Fig. 4B. When the light irradiation is stopped and the sample temperature decreases, a second transient pyrocurrent appears with a sign opposite to that at the start of irradiation. Thus, although the pyrocurrent generated at the onset and termination of light irradiation cancels out entirely when integrated over time, the integrated dc photocurrent remains finite. The sign of this dc photocurrent reverses upon reversing the direction of electric polarization, demonstrating the terahertz BPVE via soft phonon excitation. The dc photocurrent amplitude increases toward the *T*_C_ and is abruptly suppressed above the *T*_C_ (Fig. 4C), being consistent with the trend observed in Fig. 2C. The Glass coefficient is ~5.9 × 10^−7^ cm V^−1^, which agrees well with that obtained from the pulse-excitation measurement (1.36 × 10^−6^ cm V^−1^ at 0.4 THz; see also Fig. 4A). Therefore, these results further corroborate our claims based on the pulsed-excitation experiments in terahertz region and establish the terahertz shift current with promising implications for future applications.

In conclusion, we observe the terahertz BPVE enhanced by the phonon excitations. The terahertz photocurrent spectroscopy demonstrates that the σ^(2)^ spectra show the resonance structures for both soft phonon and normal infrared optical phonon; the peak magnitude of σ^(2)^ is particularly enhanced for the soft phonon reflecting the strong electron-phonon coupling. These observations are never explained by the conventional mechanisms such as the photocarrier drifting for the photocurrent in *p*-*n* junction but by the extended shift current mechanism driven by phonon excitation. Unlike the conventional photocurrents, this mechanism originates from the instantaneous shift of electronic wave function by phonon excitation, being intimately related to the quantum geometric Berry connection. This interpretation is supported by the experimental observation of resonance in σ^(2)^, as theoretically predicted, and by the first-principles calculation incorporating the shift current mechanism. These findings reveal that the observation of terahertz photocurrent directly manifests the underlying electronic quantum geometry. The quantitative evaluation of photovoltaic performance elucidates high conversion efficiency for soft phonon in SbSI as compared to the electronic excitations in representative materials, partly due to the low-energy divergence characteristic of the phonon-induced shift current. The extracted terahertz Glass coefficient is substantially larger than that of BaTiO_3_ and is comparable to the large value reported for shift currents generated by electronic excitation in the Weyl semimetal TaAs. Notably, while the large shift current in TaAs appears in the mid-infrared region—long believed to be the lower frequency limit for observing the BPVE—we demonstrate a comparable figure of merit in the terahertz range. These findings demonstrate the high efficiency and broad applicability of phonon-induced shift current through the quantum geometry, which enriches the potential materials with large terahertz photoresponsivity. The quantum geometry has increasingly been recognized as a key concept for understanding and engineering unprecedented and functional electromagnetic responses. The present work clearly demonstrates a previously unknown conversion process of terahertz light to dc current through the quantum geometry, accelerating further developments of the terahertz and far-infrared technology.

## MATERIALS AND METHODS

### Single crystal growth

Single crystals of SbSI were grown by a chemical vapor transport technique. The sample size is typically 1 mm by 5 mm by 0.3 mm.

### Electric polarization measurement

The electric polarization is deduced by measuring the pyroelectric current with increasing the temperature. The single-domain state is stabilized by the field cooling from ~310 K well above *T*_C_ with application of the electric field of ±1.4 kV/cm.

### Intense terahertz pulse generation

For a light source, we used a regenerative amplified Ti:sapphire laser system with the pulse energy of 5 mJ, the pulse duration of 100 fs, the repetition rate of 1 kHz, and the center wavelength of 800 nm. The intense terahertz pulses were generated by the tilted-pulse front method with a LiNbO_3_ crystal (*31*). The spot size was about 1 mm. The time waveform of the terahertz pulse is measured by the electro-optic sampling technique with using a ZnTe (110) crystal. The intensity of the terahertz light is controlled by using the pair of wire grid polarizers. The terahertz power is estimated by using a power meter (T-RAD, GENTEC-EO).

### Optical conductivity and dielectric spectra

Linear optical conductivity and dielectric spectra in the visible region is deduced by using the Kramers-Kronig transformation of the reflectivity spectrum. The terahertz spectra for *E*^ω^||*c* and *E*^ω^⊥*c* are respectively calculated from the data shown in (*26*) and measured by using the terahertz time-domain spectroscopy (for more details, see note S1).

### Pulsed photocurrent measurement

We measured the pulsed photocurrent signal for each light pulse by using an oscilloscope and a preamplifier (DHPCA-100; Femto) with dc coupling. The bandwidth of the preamplifier was 200 MHz except for the long-time scan measurement shown in Fig. 2 (E and F), where it was 14 MHz. We set the zero level by subtracting the electrical signal without light illumination as the background. The light is focused at the center of the sample, away from the Pt electrodes fabricated by sputtering technique to eliminate the spurious effects; the Pt electrode is found to be suitable to efficiently pick up the photocurrent in the previous study (*29*). The photocurrent pulse duration is determined presumably by the bandwidth of the preamplifier (200 MHz), as indicated by the duration of the main photocurrent pulse (~5 ns). Meanwhile, we also observe the similarly shaped secondary and even tertiary pulse signals, leading to more broadened pulse. These parasitic signals are known as the ringing effect, which inevitably occurs for a short electrical pulse due to the parasitic capacitances and inductances (and impedance mismatch) in the circuit.

### CW photocurrent measurement

We measured the photocurrent signal under the CW terahertz light irradiation. We used IMPATT Diode (TeraSense) as a light source with irradiated power to the sample of ~38 μW and frequency of 96.47 GHz, and measured the current using an electrometer (Keithley 6517A).

## Supplementary Materials

### This PDF file includes:

Supplementary Text Figs. S1 to S9 References
