# Supplementary material for: Large terahertz photovoltaic effect enhanced by phonon excitations in ferroelectric semiconductor SbSI
Source: Sci Adv. 2026 Mar 6;12(10):eadw9796. doi: 10.1126/sciadv.adw9796 (PMC12965303; doi:10.1126/sciadv.adw9796)
Supplement: Supplementary file 1 — Supplementary Text Figs. S1 to S9 References [file sciadv.adw9796_sm.pdf]

Science Advances
AAAS

Supplementary Materials for

Large terahertz photovoltaic effect enhanced by phonon excitations in ferroelectric semiconductor SbSI

Yoshihiro Okamura *et al.*

Corresponding author: Yoshihiro Okamura, okamura@ap.t.u-tokyo.ac.jp;
Youtarou Takahashi, youtarou-takahashi@ap.t.u-tokyo.ac.jp

Sci. Adv. **12**, eadw9796 (2026)
DOI: 10.1126/sciadv.adw9796

This PDF file includes:

Supplementary Text
Figs. S1 to S9
References

Supplementary Text

Supplementary Note 1: Terahertz time-domain spectroscopy of the normal optical phonon polarized perpendicular to the *c* axis.

We characterized the terahertz dielectric response perpendicular to the electric polarization along the *c* axis by means of the terahertz time-domain spectroscopy. In this measurement, the laser pulses with a duration of 100 fs from a mode-locked Ti:sapphire laser were split into two paths to generate and detect terahertz light pulses using the photoconductive antenna. Figure S2A shows the time waveform for the transmitted terahertz electric field for the sample and blank; the sample thickness is ~ 300 μm. On this basis, we deduce the refractive index n and extinction coefficient κ at each temperature (Figs. S2B and S2C). We clearly observe the sharp resonance structure around 3.5 meV with little spectral change by temperature.

Supplementary Note 2: Estimation of the through rate of the pulsed photocurrent.

We used the electrical circuit used for the ultrafast photocurrent detection, which inevitably causes the attenuation and broadening of current pulses. Thus, we estimated the through rate of photocurrent pulse by comparing the photoresponsivity for the continuous-wave (CW) light and the pulse light excitations.

More specifically, we measure the photocurrent upon the electronic excitation at each excitation energy by using the optical parametric amplifier pumped by a regenerative amplified Ti:sapphire laser system, as shown in Fig. S5A. On this basis, we calculated the photon-energy dependence of photoresponsivity, which was evaluated by the integrated photocurrent, or equivalently charge accumulation for each photocurrent pulse, normalized by the absorbed pulse power (Fig. S5B, blue circles). On the other hand, the photoresponsivity for the CW excitation has been already reported in the previous study (*18*) (Fig. S5B, red curve). Then, we compared the spectral responses for these two cases, which show the quantitative coincidence when the photoresponsivity for the CW excitation is multiplied by 0.02. Thus, we deduce the through rate of the pulsed photocurrent of ~ 2 %.

Supplementary Note 3: First-principles calculation of the shift current.

We have carried out the *ab initio* calculation of the shift current conductivity caused by the phonon excitation. We first perform *ab initio* calculations of the electronic band structure, phonon band structure and electron-phonon (e-ph) coupling matrix elements of SbSI in the low temperature orthorhombic structure with space group $Pna2_1$. The electronic structure calculations are based on the density functional theory with the generalized gradient approximation (GGA) to the exchange-correlation potential (*36*), and the phonon band structure and e-ph coupling matrix element calculations are based on the density functional perturbation theory (DFPT) (*37*). The calculations are carried out using pseudopotential plane wave method, as implemented in the QUANTUM ESPRESSO program (*38*). The interaction between the valence electrons and ionic cores is treated by the norm-conserving pseudopotentials (*39*). The plane wave expansion cut-off energy is 60 Ry and the total energy convergence criterion is 10^{-9} Ry. We use the experimentally determined crystalline structure with lattice constants a = 8.5 Å, b = 10.1 Å and c = 4.05 Å. In the present self-consistent electronic band structure calculations, a k-point mesh of 4×4×8 in the Brillouin zone (BZ) is used. The phonon band structure and e-ph coupling matrix element calculations based on the DFPT (*37*) are carried out using a 2×2×4 q-point mesh in the BZ. The Gaussian broadening method with width of 0.02 Ry is used for the BZ integration.

A theoretical formulation of the phonon-driven shift current has been given in ref. 22. On the basis of this formulation, we then calculate the shift current conductivity due to the phonon excitation using the calculated electronic and phononic band structures as well as the e-ph

coupling matrix elements. Here we deal with the phonon excitation using terahertz-light radiation which has a negligibly small wave vector (i.e., $q \approx 0$), i.e., only the BZ center phonons need to be considered. In the present case, therefore, the shift current conductivity can be written as

$$\sigma_{ijk}^{(2)}(\omega) = \frac{e^3}{\hbar^3\omega^2}\Pi_{ijk}(\omega), \tag{S1}$$

where the response function is given by (*22*)

$$\Pi_{ijk}(\omega) = \frac{\pi\hbar^2}{V_c}\sum_{\nu}\mathrm{Im}\left[A_1^j(\omega)A_2^{ik}(\omega) - A_1^k(-\omega)A_2^{ij}(-\omega)\right]\delta(\omega-\omega_\nu), \tag{S2}$$

$$A_1^j(\omega) = \sum_{\mathbf{k}}\frac{1}{N_k}\sum_{mn} f_{mn}\frac{g_{mn}v_{nm}^j}{\hbar(\omega-\omega_{nm})},$$

$$A_2^{ik}(\omega) = \sum_{\mathbf{k}}\frac{1}{N_k}\left[\sum_{\substack{mn\\ l\neq m}}\frac{v_{ml}^i v_{ln}^k g_{mn}}{\hbar^2\omega_{lm})}\left(\frac{f_{mn}}{\omega_{nm}-\omega} - \frac{f_{ln}}{\omega_{nl}-\omega}\right) + \sum_{\substack{mn\\ l\neq m}}\frac{(v_{ml}^i v_{ln}^k g_{mn})^*}{\hbar^2\omega_{lm})}\left(\frac{f_{mn}}{\omega_{nm}+\omega} - \frac{f_{ln}}{\omega_{nl}+\omega}\right)\right].$$

Here m, n and l are the electronic band indices, $g_{mn} = g_{mn}(\mathbf{k})$ is the interband e-ph coupling matrix element for $q = 0$, $v_{mn}^i = v_{mn}^i(\mathbf{k})$ is the i-component of the interband velocity matrix element, $f_{mn} = f_m - f_n$ where f_m and f_n are the Fermi distribution functions, V_c is the unit cell volume, and $\hbar\omega_{mn} = \hbar\omega_m(\mathbf{k}) - \hbar\omega_n(\mathbf{k}) = \varepsilon_m(\mathbf{k}) - \varepsilon_n(\mathbf{k})$ where $\varepsilon_m(\mathbf{k})$ ($\varepsilon_n(\mathbf{k})$) is the energy of band $m(n)$ at $\boldsymbol{k}$. For a nonmagnetic material with time-reversal symmetry, $v_{mn}^i(-\mathbf{k}) = -v_{nm}^i(\mathbf{k})$ and $g_{mn}(-\mathbf{k}) = g_{nm}(\mathbf{k})$. As a result, $A_1^j(\omega)$ and $A_2^{ij}(\omega)$ are purely imaginary and real, respectively. Assuming $\omega_{nm}^2 \gg \omega_\nu^2$ and $\frac{1}{\omega_{nm}+\omega_\nu} \approx \omega_{nm}(1-\frac{\omega_\nu}{\omega_{nm}})$, we have

$A_1^j(\omega) = \sum_{\mathbf{k}}\frac{i\omega}{N_k}\sum_{mn} f_{mn}\frac{\mathrm{Im}(g_{mn}v_{nm}^j)}{\hbar\omega_{nm}^2} = i\hbar\omega B_1^j(\omega)$, and $B_1^j(\omega) = \sum_{\mathbf{k}}\frac{1}{N_{kc}}\sum_{mn} f_{mn}\frac{\mathrm{Im}[g_{mn}v_{nm}^j]}{\hbar\omega_{nm}^2}$. Similarly, we can obtain $A_2^{ij}(\omega) = \sum_{\mathbf{k}}\frac{2}{N_k}\left[\sum_{\substack{mn\\ l\neq m}}\frac{\mathrm{Re}\left[v_{ml}^i v_{ln}^j g_{mn}\right]}{\hbar^2\omega_{lm}}\left(\frac{f_{mn}}{\omega_{nm}} - \frac{f_{ln}}{\omega_{nl}}\right)\right] = 2B_2^{ij}(\omega)$, and

$$B_2^{ij}(\omega) = \sum_{\mathbf{k}}\frac{1}{N_k}\left[\sum_{\substack{mn\\ l\neq m}}\frac{\mathrm{Re}[v_{ml}^i v_{ln}^j g_{nm}]}{\hbar^2\omega_{lm}}\left(\frac{f_{mn}}{\omega_{nm}} - \frac{f_{ln}}{\omega_{nl}}\right)\right].$$

Therefore, the diagonal shift current conductivity can be written as

$$\sigma_{ijj}^{(2)}(\omega) = \frac{4\pi e^3}{\hbar\omega V_c}\sum_{\nu} B_1^j(\omega)B_2^{ij}(\omega)\delta(\omega-\omega_\nu), \tag{S3}$$

where ω_ν is the frequency of the ν phonon eigenmode.

For comparison, we have also performed an *ab initio* calculation of the phonon linear optical response, based on a theoretical formulation reported in ref. 22. Specifically, the phonon optical conductivity for a nonmagnetic semiconductor is given by

$$\sigma_{ij}^{(1)}(\omega) = -\frac{\pi e^2\hbar\omega}{V_c}\sum_{\nu}\mathrm{Im}\left[C_1^j(\omega_\nu)\right]\mathrm{Im}\left[C_1^i(\omega_\nu)\right]\delta(\omega-\omega_\nu), \tag{S4}$$

where $C_1^i(\omega) = \sum_{\mathbf{k}} \frac{1}{N_k} \left[\sum_{\substack{mn \\ l \neq m}} \frac{f_{mn} g_{mn} v_{nm}^i}{\hbar^2 \omega_{mn} (\omega_{mn} + \omega_v)} \right]$. Due to the time-reversal symmetry, $C_1^i(\omega)$ is purely imaginary.

Since a dense *k*-point mesh with a very large number of *k*-points in the Brillouin zone is needed to obtain the accurate shift-currents, we adopt the efficient Wannier function interpolation scheme to calculate the e-ph coupling and interband velocity matrix elements (*40*), as implemented in the EPW program (*41*). The Wannier orbitals used are the *p* orbitals of Sb, S and I atoms and these 36 Wannier orbitals are optimized by fitting them to the *ab initio* band structure. The *ab initio* and Wannier orbital-interpolated band dispersions are nearly identical (not shown here). We use a dense Γ-centered *k*-point mesh of 50×50×100 in the BZ integration to calculate the shift-current conductivity as well as linear optical conductivity. In the present calculations, the $\delta(\omega - \omega_v)$ is replaced by the Gaussian function with broadening width Γ = 3 meV to take the finite carrier relaxation time (τ) into account. The calculated phonon-driven shift current conductivity spectra are displayed in Fig. 3C in the manuscript and also Fig. S8. The calculated linear optical conductivity spectra are presented in Fig. S9.

The calculated shift current and linear optical conductivity spectra (Figs. S8 and S9) agree reasonably well with the corresponding experimental spectra. For example, for both the optical electric field and photocurrent along the *c*-axis, the conductivity elements *ccc* and *cc* exhibit a broad peak at photon energy of 5.0 meV and 6.2 meV, respectively (see Figs. S8 and S9). The magnitudes of the calculated *cc* linear optical conductivity agree well with the measured ones. The magnitudes of the calculated *ccc* shift current conductivity are slightly larger than the measured values. For the other two directions perpendicular to the *c*-axis, the conductivity elements are more than ten times smaller.

Supplementary Note 4: The bandwidth of terahertz BPVE.

The bandwidth of terahertz photoresponsivity is one important aspect for application of terahertz detector. The present material SbSI is highly insulating, showing large (dark) resistance on the order of gigaohms, and exhibits no resistance change under the terahertz irradiation because of no photocarrier generation. Therefore, the overall time constant of detection circuit is governed mainly by the load resistance rather than the sample resistance. Thus, the RC time constant is expected to be typically the nanosecond range, which is a key advantage of shift-current based terahertz detector.

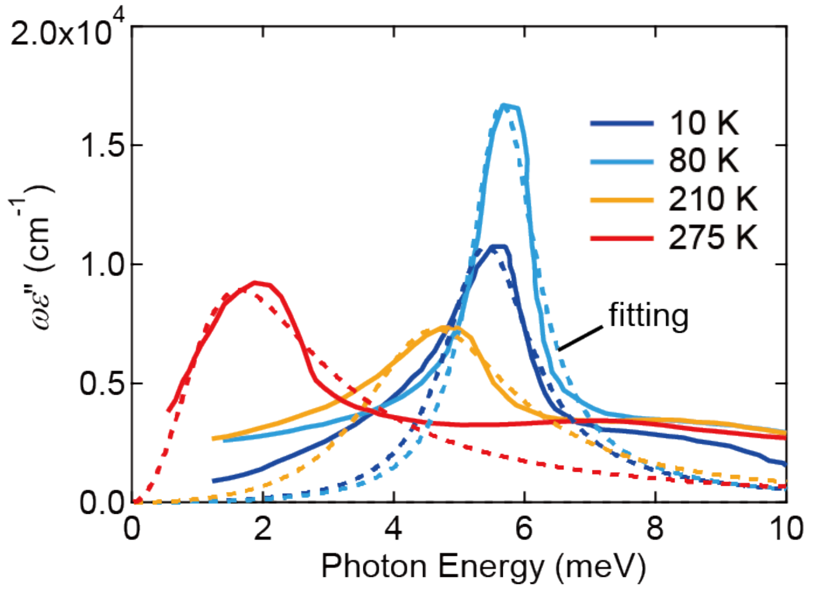

Fig. S1.

Terahertz dielectric response for the soft phonon. The imaginary part of terahertz dielectric constant for $E^{\omega}||c$ multiplied by the angular frequency at each temperature (solid curves). These data were taken from ref. 26. The dotted curves represent the fits based on the harmonic oscillator model, given as, $\varepsilon(\omega) = \frac{A}{\omega^2 - \omega_0{}^2 - i\omega\Gamma}$. The obtained ω_0 and Γ are, for example, 1.65 and 2.71 meV at 275 K, respectively. The fitting functions are used for calculation of $\sigma^{(2)}$ in Fig. 3B. Discrepancies between the experimental spectra and the fits are observed around the lower lying tail of the peaks, especially apparent at 80 K. These discrepancies would be partly caused by the inevitable uncertainty in Kramers-Kronig analysis used in the early work; the uncertainty of Kramers-Kronig transformation is always enlarged around the edge of experimental energy window. The estimation of $\sigma^{(2)}$ is reliable in the vicinity of the resonance frequency, but its accuracy will decrease in the lower-energy region.

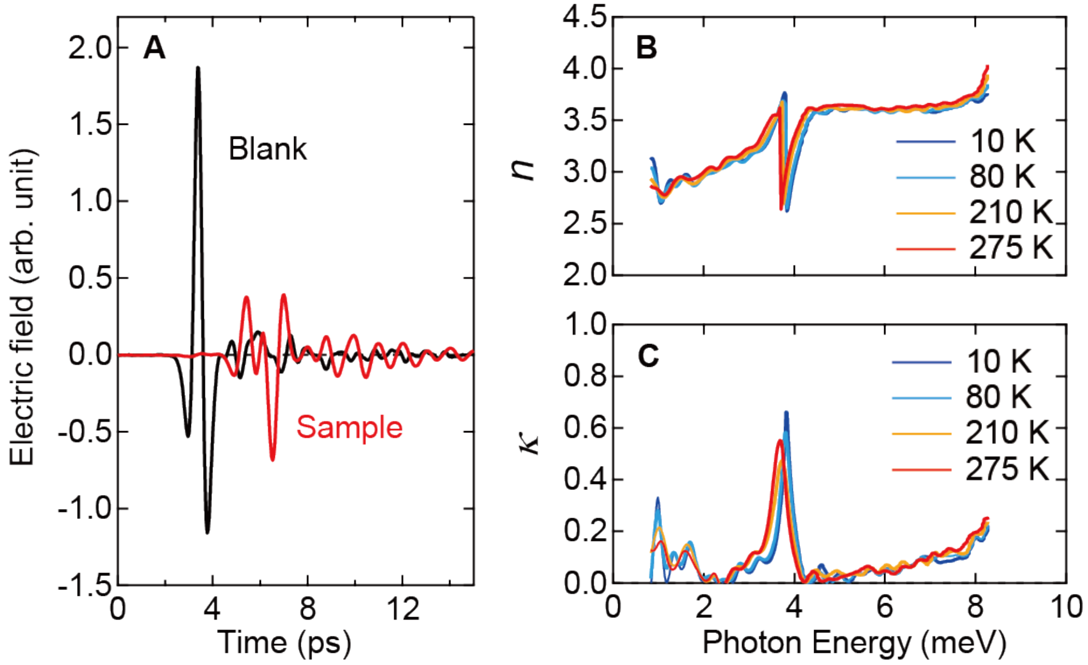

Fig. S2.

Terahertz time-domain spectroscopy polarized perpendicular to the *c* axis. (**A**) Time waveform of transmitted terahertz electric field for the sample (red curve) and blank (black curve). (**B**,**C**) The refractive index n (**B**) and extinction coefficient κ (**C**) at each temperature.

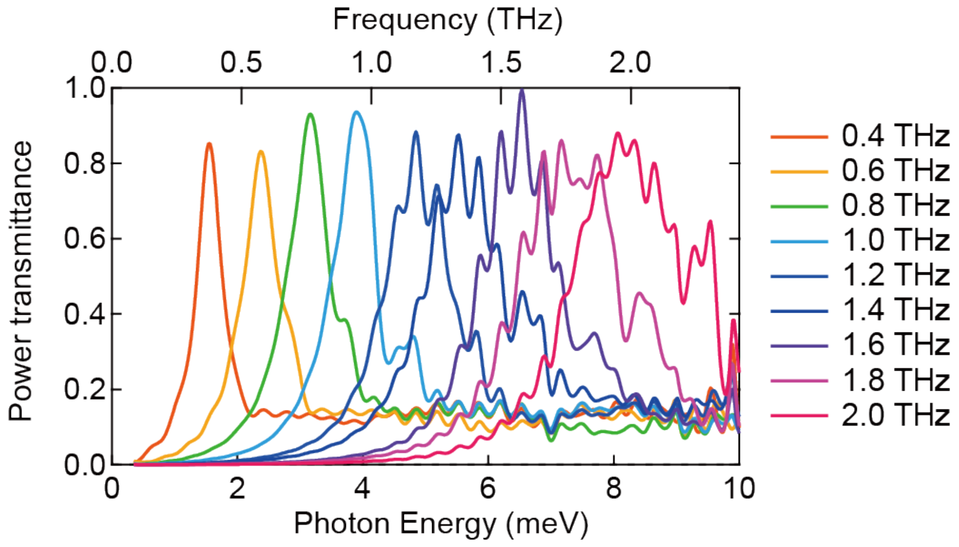

Fig. S3.

Power transmittance of the terahertz band pass filter. The power transmittance spectrum for each filter was measured by the terahertz time-domain spectroscopy. The presently used bandpass filters with punching hole mesh always show leakage in high frequency region, because the high frequency terahertz wave with short wavelength can pass through the punching hole due to the diffraction limit.

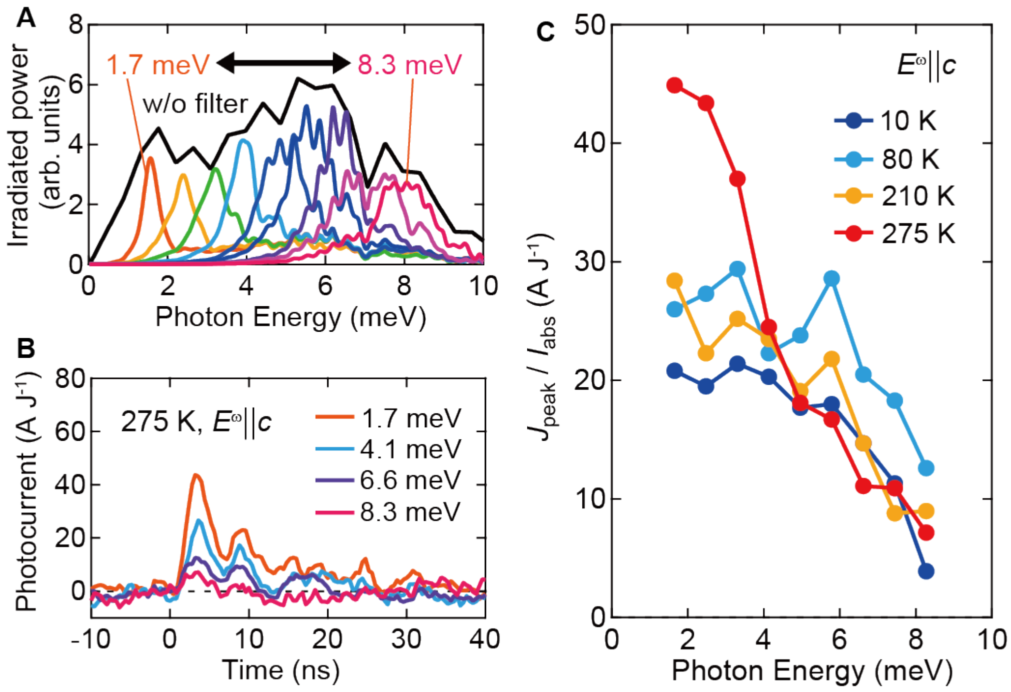

Fig. S4.

Spectral response of the phonon-induced shift current. (**A**) The incident terahertz power spectrum without and with the bandpass filters. The black curve corresponds to the spectrum without the filters. The colored curves denote the power spectra with each bandpass filter. The filtering energy is chosen from 1.65 meV (0.4 THz) to 8.26 meV (2 THz) every 0.826 meV (0.2 THz). (**B**) The example of time waveforms of the photocurrent normalized by the irradiated power at each filtered frequency. (**C**) The photocurrent response J_{peak} normalized by the absorbed power I_{abs}. I_{abs} is calculated by considering the product of the energy spectrum of irradiated terahertz light and absorption coefficient. $J_{\mathrm{peak}}/I_{\mathrm{abs}}$ roughly corresponds to $\sigma^{(2)}/\sigma^{(1)} = \frac{e}{\hbar\omega}R$ as suggested by comparison of Eqs. (1) and (2) in main text, and therefore, this relation predicts the low-energy enhancement of $J_{\mathrm{peak}}/I_{\mathrm{abs}}$ through $\hbar\omega$ in denominator.

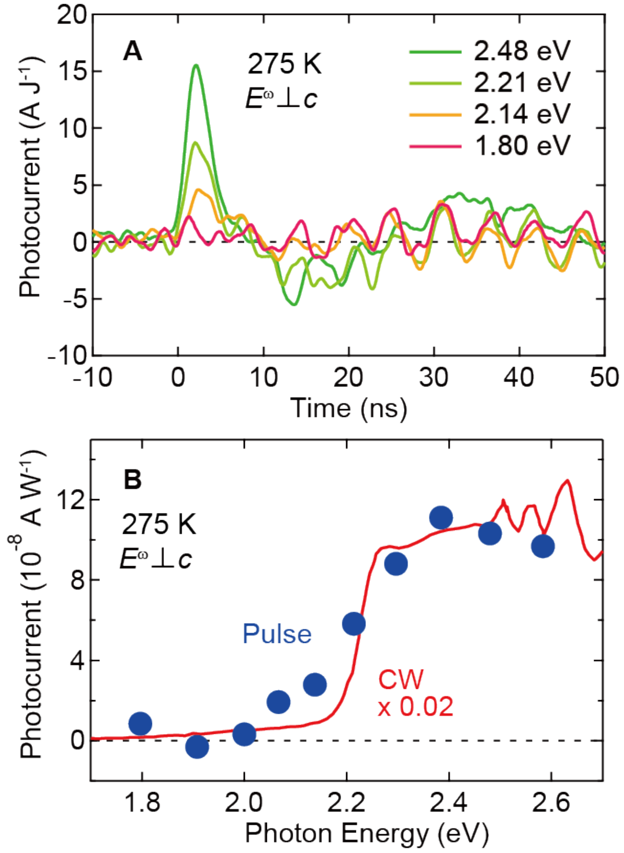

Fig. S5.

Estimation of the through rate of the pulse photocurrent. (**A**) The example of time waveforms of the photocurrent normalized by the irradiated power at each photon energy. (**B**) The photocurrent response for the CW excitation (red curve) and pulse excitation (blue circles). The spectrum for the CW excitation is calculated from the previous study (*18*). When the photoresponsivity for the CW excitation is multiplied by 0.02, the spectra for those two conditions quantitatively coincide with each other. Therefore, the through rate of photocurrent pulse is estimated to be 0.02.

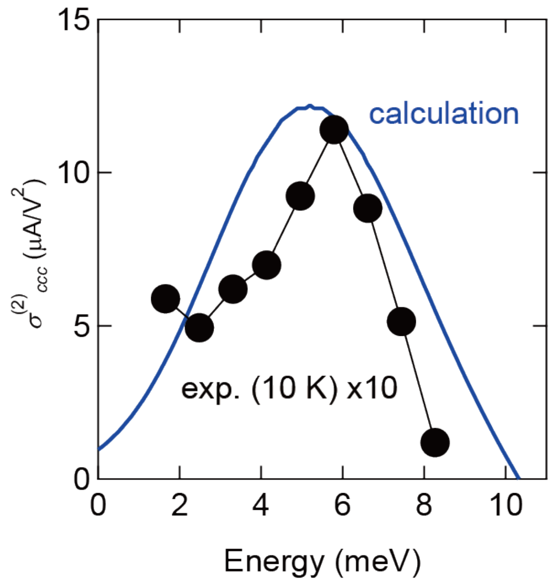

Fig. S6.

Direct comparison of experimental and theoretical nonlinear conductivities.
Since the calculation considers the zero-temperature limit, we show the direct comparison to the experimental spectrum at 10 K, demonstrating the semi-quantitative agreement. Meanwhile, the quantitative agreement of $\sigma^{(2)}$ regarding peak height and its temperature dependence remains to be investigated in future work.

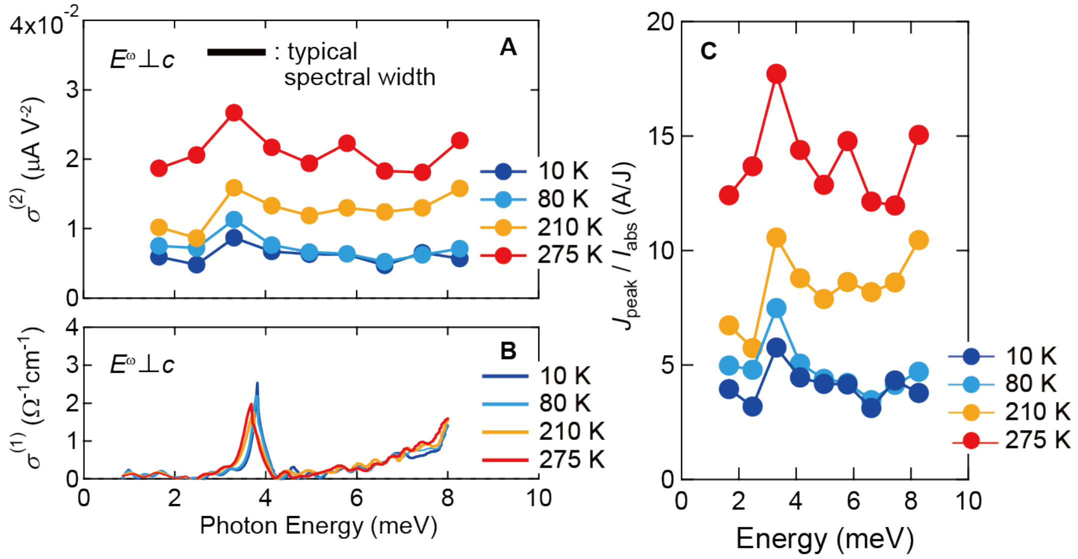

Fig. S7.

Nonlinear optical conductivity of the infrared-active optical phonon. (**A**) Nonlinear optical conductivity $\sigma^{(2)}$ for $E^{\omega}\perp c$. The black horizontal bar represents the typical half width at half maximum of the irradiated terahertz power spectrum. (**B**) Linear optical conductivity $\sigma^{(1)}$ for $E^{\omega}\perp c$. We discern the resonance peak in the $\sigma^{(2)}$ spectra derived from the infrared-active optical phonon polarized perpendicular to the *c* axis. The spectral characteristics of $\sigma^{(2)}$ strongly reflects the phonon resonance in $\sigma^{(1)}$ but also should show some deviation from $\sigma^{(1)}$, as suggested by Eq. (2) in main text. The shift vector *R* is a frequency dependent quantity, and $\hbar\omega$ in denominator in Eq. (2) also affects the spectral shape of $\sigma^{(2)}$. We note that the *R* can be expressed in terms of simple band parameters within the toy model discussed in our previous work (*22*). However, it is natural to expect that *R* acquires a frequency dependence in real materials due to complex band dispersions and the resulting electron–phonon interactions. On the other hand, the peak width in experimentally obtained $\sigma^{(2)}$ for $E^{\omega}\perp c$ might be more broadened than the actual width because the spectral width of this phonon mode in $\sigma^{(1)}$ is shaper than that of the terahertz band pass filter. The flat background in $\sigma^{(2)}$ is explained by the shift current from dielectric loss (*42*); the lower-lying tail of phonon resonance can exhibit the photocurrent below the resonance frequency of phonon. (**C**) The photon energy dependence of J_{peak}/I_{abs} for $E\perp c$. The pronounced anisotropy between $\sigma^{(2)}{}_{ccc}$ and $\sigma^{(2)}{}_{caa}$ (a difference of nearly two orders of magnitude) cannot be attributed solely to the anisotropy in $\sigma^{(1)}$. The $\sigma^{(2)}/\sigma^{(1)}$ at the soft phonon energy ($E\|c$) is 8x10^{-7} cm/V, while that at the normal phonon energy ($E\perp c$) is 1.4x10^{-8} cm/V. The enhanced anisotropy in $\sigma^{(2)}$ suggests the strong mode dependence of shift vector (see Eq. (2) in main text).

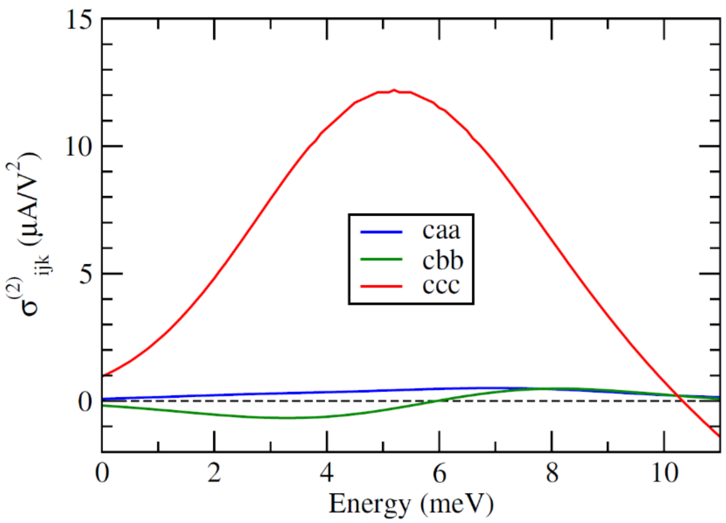

Fig. S8.

Calculation of nonlinear optical conductivity. The *ccc* component represents the photocurrent response along the *c* axis for the soft phonon polarized along *c* axis. The *caa* and *cbb* components correspond to the infrared active phonon polarized perpendicular to *c* axis.

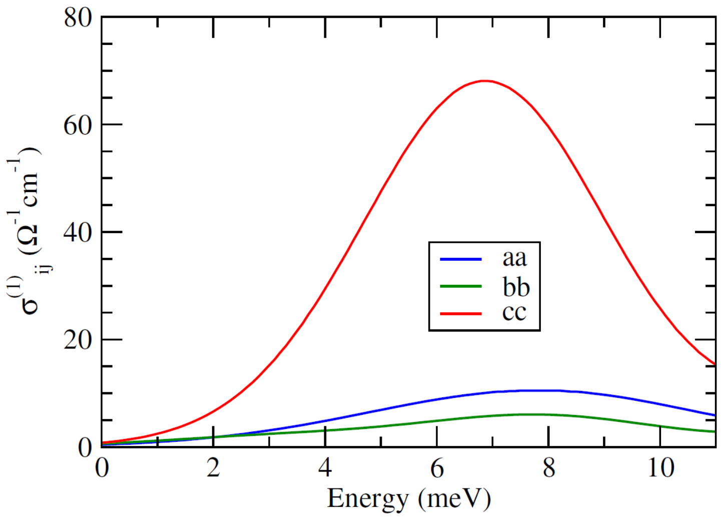

Fig. S9.

Calculation of linear optical conductivity. The *cc* component represents the optical conductivity for the soft phonon polarized along *c* axis. The *aa* and *bb* components correspond to the infrared active phonon polarized perpendicular to *c* axis.

REFERENCES

1. M. Tonouchi, Cutting-edge terahertz technology. *Nat. Photonics* **1,** 97–105 (2007).

2. F. Sizov, A. Rogalski, THz detectors. *Prog. Quantum Electron.* **34,** 278–347 (2010).

3. A. Rogalski, Progress in focal plane array technologies. *Prog. Quantum Electron.* **36,** 342–473 (2012).

4. L. Vicarelli, M. S. Vitiello, D. Coquillat, A. Lombardo, A. C. Ferrari, W. Knap, M. Polini, A. Tredicucci, Graphene field-effect transistors as room-temperature terahertz detectors. *Nat. Mater.* **11,** 865–871 (2012).

5. F. H. L. Koppens, T. Mueller, Ph. Avouris, A. C. Ferrari, M. S. Vitiello, M. Polini, Photodetectors based on graphene, other two-dimensional materials and hybrid systems. *Nat. Nanotechnol.* **9,** 780–793 (2014).

6. Q. Ma, A. G. Grushin, K. S. Burch, Topology and geometry under the nonlinear electromagnetic spotlight. *Nat. Mater.* **20,** 1601–1614 (2021).

7. Y. Tokura, N. Nagaosa, Nonreciprocal responses from non-centrosymmetric quantum materials. *Nat. Commun.* **9**, 3740 (2018).

8. N. Nagaosa, Y. Yanase, Nonreciprocal transport and optical phenomena in quantum materials. *Annu. Rev. Condens. Matter Phys.* **15,** 63–83 (2024).

9. T. Morimoto, N. Nagaosa, Topological nature of nonlinear optical effects in solids. *Sci. Adv.* **2,** e1501524 (2016).

10. B. I. Sturman, V. M. Fridkin, The photovoltaic and photorefractive effects in noncentrosymmetric materials (Gordon and Beach Science Publishers, 1992).

11. J. E. Sipe, A. I. Shkrebtii, Second-order optical response in semiconductors. *Phys. Rev. B* **61,** 5337–5352 (2000).

12. S. M. Young, A. M. Rappe, First principles calculation of the shift current photovoltaic effect in ferroelectrics. *Phys. Rev. Lett.* **109,** 116601 (2012).

13. L. Z. Tan, F. Zheng, S. M. Young, F. Wang, S. Liu, A. M. Rappe, Shift current bulk photovoltaic effect in polar materials-hybrid and oxide perovskites and beyond. *npj Comput. Mater.* **2,** 16026 (2016).

14. M. Nakamura, S. Horiuchi, F. Kagawa, N. Ogawa, T. Kurumaji, Y. Tokura, M. Kawasaki, Shift current photovoltaic effect in a ferroelectric charge-transfer complex. *Nat. Commun.* **8,** 281 (2017).

15. G. B. Osterhoudt, L. K. Diebel, M. J. Gray, X. Yang, J. Stanco, X. Huang, B. Shen, N. Ni, P. J. W. Moll, Y. Ran, K. S. Burach, Colossal mid-infrared bulk photovoltaic effect in a type-I Weyl semimetal. *Nat. Mater.* **18,** 471–475 (2019).

16. N. Ogawa, M. Sotome, Y. Kaneko, M. Ogino, Y. Tokura, Shift current in the ferroelectric semiconductor SbSI. *Phys. Rev. B* **96,** 241203 (2017).

17. M. Sotome, M. Nakamura, J. Fujioka, M. Ogino, Y. Kaneko, T. Morimoto, Y. Zhang, M. Kawasaki, N. Naogasa, Y. Tokura, N. Ogawa, Spectral dynamics of shift current in ferroelectric semiconductor SbSI. *Proc. Natl. Acad. Sci. U.S.A.* **116,** 1929–1933 (2019).

18. H. Hatada, M. Nakamura, M. Sotome, Y. Kaneko, N. Ogawa, T. Morimoto, Y. Tokura, M. Kawasaki, Defect tolerant zero-bias topological photocurrent in a ferroelectric semiconductor. *Proc. Natl. Acad. Sci. U.S.A.* **117,** 20411–20415 (2020).

19. T. Morimoto, N. Nagaosa, Topological aspects of nonlinear excitonic processes in noncentrosymmetric crystals. *Phys. Rev. B* **94,** 035117 (2016).

20. T. Morimoto, N. Nagaosa, Shift current from electromagnon excitations in multiferroics. *Phys. Rev. B* **100,** 235138 (2019).

21. M. Sotome, M. Nakamura, T. Morimoto, Y. Zhang, G.-Y. Guo, M. Kawasaki, N. Nagaosa, Y. Tokura, N. Ogawa, Terahertz emission spectroscopy of ultrafast exciton shift current in the noncentrosymmetric semiconductor CdS. *Phys. Rev. B* **103,** L241111 (2021).

22. Y. Okamura, T. Morimoto, N. Ogawa, Y. Kaneko, G.-Y. Guo, M. Nakamura, M. Kawasaki, N. Nagaosa, Y. Tokura, Y. Takahashi, Photovoltaic effect by soft phonon excitation. *Proc. Natl. Acad. Sci. U.S.A.* **119,** e2122313119 (2022).

23. M. Ogino, Y. Okamura, K. Fujiwara, T. Morimoto, N. Nagaosa, Y. Kaneko, Y. Tokura, Y. Takahashi, Terahertz photon to dc current conversion via magnetic excitations of multiferroics. *Nat. Commun.* **15,** 4699 (2024).

24. E. Fatuzzo, G. Harbeke, W. J. Merz, R. Nitsche, H. Roetschi, W. Ruppel, Ferroelectricity in SbSI. *Phys. Rev.* **127,** 2036 (1962).

25. A. Kikuchi, Y. Oka, E. Sawaguchi, Crystal structure determination of SbSI. *J. Physical Soc. Japan* **23,** 337–354 (1967).

26. D. K. Agrawal, C. H. Perry, Long-wavelength optical phonons and phase transitions in SbSI. *Phys. Rev. B* **4,** 1893–1902 (1971).

27. R. Nitsche, W. J. Merz, Photoconduction in ternary V-VI-VII compounds. *J. Phys. Chem. Solid* **13,** 154–155 (1960).

28. V. M. Fridkin, Photoferroelectrics (Springer, 1979), 10.1007/978-3-642-81351-1.

29. M. Nakamura, H. Hatada, Y. Kaneko, N. Ogawa, Y. Tokura, M. Kawasaki, Impact of electrodes on the extraction of shift current from a ferroelectric semiconductor SbSI. *Appl. Phys. Lett.* **113,** 232901 (2018).

30. M. Nakamura, H. Hatada, Y. Kaneko, N. Ogawa, M. Sotome, Y. Tokura, M. Kawasaki, Non-local photocurrent in a ferroelectric semiconductor SbSI under local photoexcitation. *Appl. Phys. Lett.* **116,** 122902 (2020).

31. J. Hebling, G. Almasi, I. Z. Kozma, J. Kuhl. Velocity matching by pulse front tilting for large area THz-pulse generation. *Opt. Express* **10,** 1161–1166 (2002).

32. A. M. Glass, D. von der Linde, T. J. Negran, High-voltage bulk photovoltaic effect and the photorefractive process in $LiNbO_3$. *Appl. Phys. Lett.* **25,** 233 (1974).

33. A. Zenkevich, Y. Matveyev, K. Maksimova, R. Gaynutdinov, A. Tolstikhina, V. Fridkin, Giant bulk photovoltaic effect in thin ferroelectric $BaTiO_3$ films. *Phys. Rev. B* **90,** 161409 (2014).

34. S. M. Young, F. Zheng, A. M. Rappe, First-principles calculation of the bulk photovoltaic effect in bismuth ferrite. *Phys. Rev. Lett.* **109,** 236601 (2012).

35. F. Zheng, H. Takenaka, F. Wang, N. Z. Koocher, A. M. Rappe, First-principles calculation of the bulk photovoltaic effect in $CH_3NH_3PbI_3$ and $CH_3NH_3PbI_{3-x}Cl_x$. *J. Phys. Chem. Lett.* **6,** 31 (2015).

36. J. P. Perdew, K. Burke, M. Ernzerhof, Generalized gradient approximation made simple. *Phys. Rev. Lett.* **77**, 3865–3868 (1996).

37. S. Baroni, S. de Gironcoli, S. Dal Corso, P. Giannozzi, Phonons and related crystal properties from density-functional perturbation theory. *Rev. Mod. Phys.* **73**, 515 (2001).

38. P. Giannozzi, O. Andreussi, T. Brumme, O. Bunau, M. Buongiorno Nardelli, M. Calandra, R. Car, C. Cavazzoni, D. Ceresoli, M. Cococcioni, N. Colonna, I. Carnimeo, A. Dal Corso, S. de Gironcoli, P. Delugas, R. A. DiStasio Jr, A. Ferretti, A. Floris, G. Fratesi, G. Fugallo, R. Gebauer, U. Gerstmann, F. Giustino, T. Gorni, J. Jia, M. Kawamura, H.-Y. Ko, A. Kokalj, E. Küçükbenli, M. Lazzeri, M. Marsili, N. Marzari, F. Mauri, N. L. Nguyen, H.-V. Nguyen, A. Otero-de-la-Roza, L. Paulatto, S. Poncé, D. Rocca, R. Sabatini, B. Santra, M. Schlipf, A. P. Seitsonen, A. Smogunov, I. Timrov, T. Thonhauser, P. Umari, N. Vast, X. Wu, S. Baroni, Advanced capabilities for materials modelling with Quantum ESPRESSO. *J. Phys. Condens. Matter* **29**, 465901 (2017).

39. D. R. Hamann, Optimized norm-conserving Vanderbilt pseudo potentials. *Phys. Rev. B* **88**, 085117 (2013).

40. F. Giustino, M. L. Cohen, S. G. Louie, Electron-phonon interaction using Wannier functions. *Phys. Rev. B* **76**, 165108 (2007).

41. S. Poncé, E. R. Margine, C. Verdi, F. Giustino, EPW: Electron-phonon coupling, transport and superconducting properties using maximally localized Wannier functions. *Comp. Phys. Commun.* **209**, 116 (2016).

42. T. Morimoto, N. Nagaosa, Direct current generation by dielectric loss in ferroelectrics. *Phys. Rev. B* **110,** 045129 (2024).
